# 1-1-12 One-Step Wash-In Scheme for Desflurane-Nitrous Oxide Low-Flow Anesthesia: Rapid and Predictable Induction

**DOI:** 10.1155/2014/867504

**Published:** 2014-06-04

**Authors:** Thepakorn Sathitkarnmanee, Sirirat Tribuddharat, Chakthip Suttinarakorn, Duangthida Nonlhaopol, Maneerat Thananun, Wilawan Somdee, Sunchai Theerapongpakdee

**Affiliations:** Department of Anesthesiology, Faculty of Medicine, Khon Kaen University, Khon Kaen 40002, Thailand

## Abstract

*Background*. We propose a 1-1-12 wash-in scheme for desflurane-nitrous oxide (N_2_O) low-flow anesthesia. The objective of our study was to determine the time to achieve alveolar concentration of desflurane (F_A_D) at 1, 2, 3, 4, 5, and 6%. *Methods*. We enrolled 106 patients scheduled for elective surgery under general anesthesia. After induction and intubation, wash-in was started with a fresh gas flow (FGF) of N_2_O : O_2_ 1 : 1 L min^−1^ and vaporizer concentration of desflurane (FD) of 12%. Ventilation was controlled to maintain P_A_CO_2_ at 30–35 mmHg. *Results*. The F_A_D rose rapidly from 0 to 4% in 2 min in a linear manner in 0.5 min increments. An F_A_D of 6% was achieved in 4 min in a linear fashion from F_A_D of 4% but in 1 min increments. An F_A_D of 1 to 6% occurred at 0.6, 1, 1.5, 2, 3, and 4 min. Heart rate during wash-in showed a statistically, albeit not clinically, significant pattern of increase. By contrast, blood pressure slightly decreased during this period. *Conclusions*. We developed a 1-1-12 wash-in scheme using a FGF of N_2_O : O_2_ 1 : 1 L min^−1^ and FD of 12% for desflurane-nitrous oxide low-flow anesthesia. A respective F_A_D of 1, 2, 3, 4, 5, and 6% can be expected at 0.6, 1, 1.5, 2, 3, and 4 min.

## 1. Introduction

The benefits of low-flow anesthesia (LFA; fresh gas flow ≤ 1 L min^−1^) include its economy, lower pollution, and conservation of heat and humidity [1]. Desflurane is well suited for LFA because it has low tissue solubility and there is no limitation of minimal fresh gas flow (FGF) even with older CO_2_ absorbers [2]. LFA needs an initial high FGF with high vaporizer concentration of desflurane (FD) in order to rapidly achieve the required concentration in the circle circuit: this is the wash-in phase [3] and many wash-in schemes have been reported. Some are complicated with multiple stages while others are simple single-step adjustments, but most need a very high FGF while achieving only some specific targets for inspired concentration (F_I_D) and alveolar concentration of desflurane (F_A_D) [2, 4, 5]. A scheme exists that can predict the entire FGF-FD combination for any F_A_D target, but it requires a complex empirical logistic regression equation and a computer program to calculate, thereby making it impracticable [6]. We propose a simple 1-1-12 wash-in scheme—a single step using FGF of N_2_O : O_2_ 1 : 1 L min^−1^ and FD of 12%—that will enable the anesthesiologist to anticipate the time needed to rapidly achieve every F_A_D target (i.e., from 1 to 6%).

The aim of this study was to determine the time to achieve F_A_D at 1, 2, 3, 4, 5, and 6% using the 1-1-12 wash-in scheme.

## 2. Materials and Methods

The current study was approved by the Institutional Review Board of Khon Kaen University (HE561247) and it was registered at http://www.clinicaltrials.gov (NCT01348984). All patients gave written informed consent before recruitment.

Our study was a descriptive trial. We calculated the sample size from a pilot study on 10 patients, which identified a standard deviation of 40 sec at F_A_D of 6%. With the total width of the expected confidence interval of 16 sec and a significance criterion of 0.05, the total number of patients required was 96. To cover a 10% dropout, 106 patients were recruited. We included patients between 18 and 64 years of age, having an ASA physical status of I or II and scheduled for elective surgery under general anesthesia with endotracheal intubation and controlled ventilation. We excluded patients with pulmonary or cardiac disease, a BMI > 30 kg m^−2^, and any contraindication of use of succinylcholine and nitrous oxide.

All patients received standard intraoperative monitoring and care. The monitoring consisted of ECG, pulse oximeter, noninvasive blood pressure, capnography, and anesthetic gas analysis. The combined anesthetic machine and gas analyzer used in this study was the Dräger Primus (Dräger AG, Lübeck, Germany). We used a standard circle circuit with a soda lime absorber. Blood pressure and heart rate were recorded before induction for a baseline. After injecting the premedication intravenous fentanyl of 1 *μ*g kg^−1^, the patient was induced with propofol at 2 mg kg^−1^. Endotracheal intubation was facilitated with succinylcholine at 1.5 mg kg^−1^. After the correct position of the tube was confirmed, ventilation was controlled using a FGF of N_2_O : O_2_ 1 : 1 L min^−1^ and FD of 12%. The tidal volume was initially set at 7 mL kg^−1^ at a respiratory rate of 12 min^−1^ and then adjusted to keep the P_A_CO_2_ around 30–35 mmHg. We recorded (a) the time to achieve F_A_D at 1, 2, 3, 4, 5, and 6%, (b) F_I_D, (c) blood pressure, and (d) heart rate. After F_A_D 6% was achieved, the FGF of N_2_O : O_2_ was reduced to 0.5–1.0 L min^−1^ and the FD was adjusted according to the judgment of the anesthesiologist.

Statistical analyses were performed using SPSS for Windows 16.0. The continuous demographic data are presented as means ± SD and the categorical as the number of patients (percentage). The primary outcome was presented as a mean ± SD and 99% confidence interval (CI). The blood pressure and heart rate at different times were compared using repeated measures analysis of variance (*r*ANOVA), *P* < 0.05 was considered statistically significant.

## 3. Results

One hundred and six patients completed the study. The demographic data of the patients are presented in Table 1.

**Figure 1:**
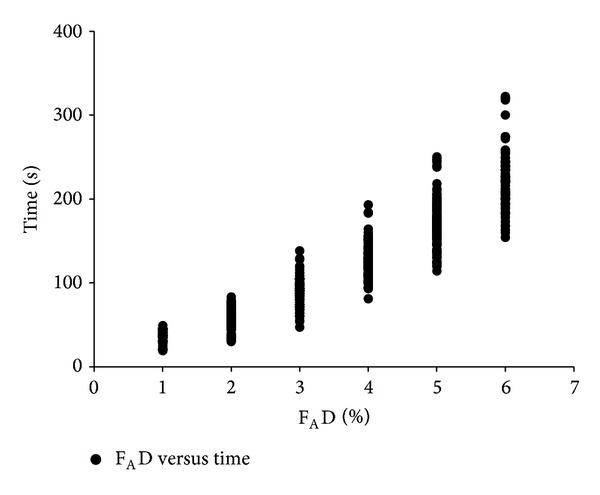
The trajectories of F_A_D versus time to achieve each F_A_D during wash-in. F_A_D =* alveolar concentration of desflurane*.

**Figure 2:**
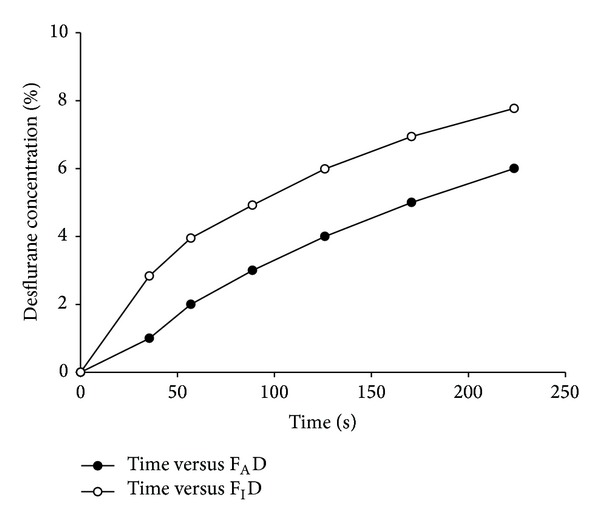
Rising pattern of F_A_D and F_I_D during wash-in. F_A_D =* alveolar concentration of desflurane; *F_I_D =* inspired concentration of desflurane*.

The trajectories of time to achieve each F_A_D for every patient during wash-in are presented in Figure 1. The time to achieve F_A_D 1 to 6% and the 99% CIs are presented in Table 2. We converted the mean time in seconds into an approximate number of minutes for practical application. The F_A_D rose rapidly and linearly from 0 to 4% within 2 min at 0.5 min increments. An F_A_D of 6% was achieved within 4 min at 1 min linear increments from an F_A_D of 4%. We could thus expect to achieve every F_A_D of 1 to 6% at 0.6, 1, 1.5, 2, 3, and 4 min. F_I_D followed in a similar pattern (Figure 2).

**Figure 3:**
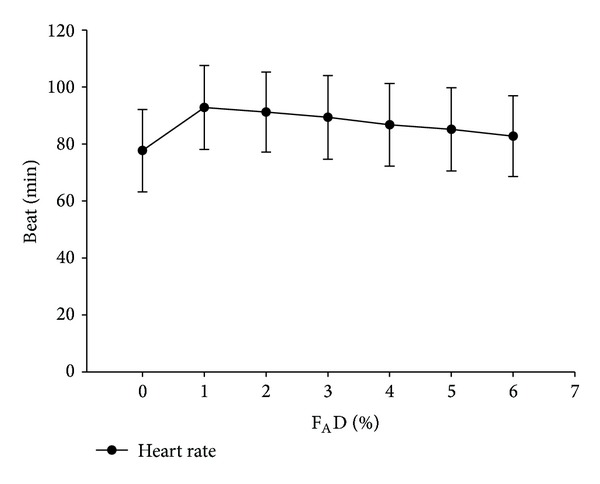
Heart rate at different F_A_D during wash-in. *P* < 0.001. F_A_D =* alveolar concentration of desflurane*.

**Figure 4:**
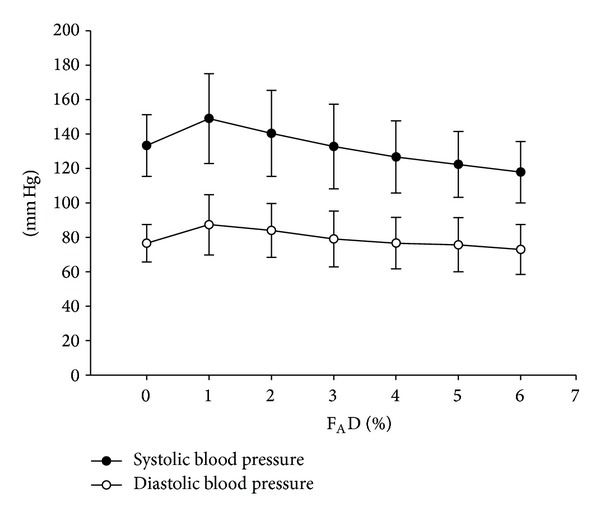
Blood pressure at different F_A_D during wash-in. *P* < 0.001. F_A_D =* alveolar concentration of desflurane*.

Heart rate during wash-in showed a statistically (but not clinically) significant increase (Figure 3). In contrast, blood pressure slightly decreased during this period (Figure 4).

## 4. Discussion

The purpose of induction and maintenance of inhalation anesthesia is to bring the patient from Guedel's classification of anesthesia stage 1 to stage 3—where the patient are to be maintained—as rapidly as possible to avoid the unpleasant phenomena of stage 2. With current intravenous drugs—for example, propofol, thiopental, and etomidate—the onset is rapid (within one minute) by rapid first compartment distribution. By contrast, current modern inhalation anesthetics—for example, isoflurane, sevoflurane, and desflurane—require a longer time to achieve a minimum alveolar concentration (MAC) with high FGF and even much longer with LFA. LFA is gaining in popularity because of its many benefits—that is, economic benefit, less pollution, conservation of heat and humidity, and availability of modern anesthesia machines and monitors. With LFA, less inhalation anesthetic is delivered into the circle circuit, so the F_I_D remains low and it takes more time to rise than using a high FGF. Thus, LFA needs an initial high FGF or low FGF with the FD set close to the maximal setting in order to shorten the time needed to achieve a therapeutic level, called the wash-in phase [3]. This wash-in phase should be accomplished within 5 min because this period covers (1) the anesthesia circuit wash-in; (2) the functional residual capacity wash-in; (3) early uptake by vessel-rich group tissues; and (4) the waning effects of intravenous induction agents [6].

Baum et al. developed a dosing scheme using N_2_O of 60–70% in O_2_ at FGF of 4.4 L min^−1^ plus FD in the range of 3.4 to 8.7%, which resulted in the F_A_D reaching values in the range of 90–95% of the fresh gas concentration within 10–15 min [2]. Mapleson created a model representing components of the breathing system, a three-compartment lung and a multicompartment representation of the patient's tissue and circulation for a 70 kg “standard man” and demonstrated that, with a FGF of O_2_ 3.5 L min^−1^ and a FD at 3 MAC, an F_A_D of 1 MAC could be achieved within 1 min [4]. This model was tested in real patients and it was found that the F_A_D exceeded 1 MAC by 2 min and remained above this value throughout the study [7–9]. Hendrickx et al. subsequently reported a single-step wash-in with N_2_O : O_2_ 4 : 2 L min^−1^ with FD of 6.5% for 15 min that could achieve an F_A_D of 4.5% [5]. The aforementioned schemes use a very high FGF (range: 3.5 to 6 L min^−1^) with an FD of between 0.5 and 3 MAC, which can only achieve a few of the specified F_A_D targets. Hendrickx et al. later proposed an empirical logistic regression model for predicting the entire range of FGF/FD combinations with clinically acceptable accuracy, from a FGF as low as 1 L min^−1^, for attaining the target F_A_D within 5 min [6]. The equation, however, is complicated and requires a computer program to do the calculation, making it impractical for daily use.

F_I_D—the effect of FGF and FD—can be increased by using a very high FGF (>3.5 L min^−1^) with a moderate FD or a low range of high FGF (2 L min^−1^) with a high FD. We developed a wash-in scheme, adapted from the logistic regression model of Hendrickx et al. [6], using a single-step FGF of only 2 L min^−1^ with an FD of 12% (approximately 2 MAC), resulting in a rapid rise of the F_I_D. From visual inspection of the trajectories of time to achieve each F_A_D in Figure 1, this scheme has acceptable intrasubject and intersubject variability. Our scheme can achieve those targets earlier at much lower FGF compared with the aforementioned schemes. We included N_2_O in our scheme because of its second gas effect [10] and additive effect on decreasing MAC of inhalation anesthetics [11]. Our scheme is practicable and yields a rapid wash-in and a prediction for achieving each F_A_D from 1 to 6%, which is 2-MAC equivalent with 50% N_2_O, at 0.6, 1, 1.5, 2, 3, and 4 min. This range covers the F_A_D required for both balanced and pure inhalation anesthesia. The fact that this scheme can achieve F_A_D of 6% within 4 min makes it ideal for inhalation anesthesia initiation without hyperexcitation. After achieving the required specific target, one can reduce the FGF to LFA range of 0.5 to 1 L min^−1^ and maintain the target F_A_D by simply setting the FD above the target by 1-2% [3, 12].

Although we found a significantly increased heart rate and decreased blood pressure during the wash-in period, the magnitude is without clinical significance as noted by Warltier and Pagel [13]. By contrast, Ebert and Muzi reported that titration of desflurane from 1 to 1.5 MAC—following thiopental induction—resulted in sympathoexcitation, hypertension, and tachycardia in healthy volunteers [14]. Such excitation may be caused by the slow increase of F_A_D to a target lower than the concentration required to block autonomic reflexes to nociceptive stimuli (MAC_BAR_) of desflurane of 1.5 to 1.7 MAC, such slow increase of F_A_D keeps the patient in the second stage longer. Our scheme results in a rapid wash-in, thereby passing the MAC_BAR_ of desflurane earlier and maintaining the patient in the surgical stage throughout the study without sympathetic overactivity.

Since our scheme uses N_2_O as part of the carrier gas, this scheme may not be inferred to the cases contraindicating the use of nitrous oxide. This scheme may have a limitation for use in obese patients since we excluded patients with a BMI > 30 kg m^−2^. Further study for such situations is required.

## 5. Conclusions

We developed a 1-1-12 wash-in scheme using a simple, single-step FGF of N_2_O : O_2_ 1 : 1 L min^−1^ and FD of 12% for desflurane-nitrous oxide low-flow anesthesia in patients requiring general anesthesia with endotracheal intubation and controlled ventilation. A respective F_A_D of 1, 2, 3, 4, 5, and 6% can be rapidly expected at 0.6, 1, 1.5, 2, 3, and 4 min. This technique proved practicable and it covered the F_A_D required for both balanced and pure inhalation anesthesia; there was a nonclinically significant increase in heart rate and decrease in blood pressure during the wash-in period.

## Figures and Tables

**Table 1:** Demographic data for the 106 patients.

Parameter	Value
Age (yr)	46.66 ± 10.20
Weight (kg)	58.36 ± 10.54
Height (cm)	159.56 ± 8.81
Sex:	
Male	43 (40.6%)
Female	63 (59.4%)
ASA classification:	
I	62 (58.5%)
II	44 (41.5%)

Data for age, weight, and height are presented as means ± SD. Sex and ASA classification are presented as a number of patients (%). ASA = American Society of Anesthesiologists.

**Table 2:** F_I_D and time at different end points of F_A_D.

	F_I_D (%)	Time (sec)	99% CI (sec)	Approximate mean time (min)
F_A_D of 1%	2.76 ± 0.59	35.42 ± 7.30	33.56–37.28	**0.6**
F_A_D of 2%	3.79 ± 0.48	56.89 ± 11.20	54.04–59.74	**1.0**
F_A_D of 3%	4.95 ± 0.36	88.63 ± 16.66	84.38–92.88	**1.5**
F_A_D of 4%	5.94 ± 0.29	126.04 ± 19.78	121.00–131.08	**2.0**
F_A_D of 5%	6.91 ± 0.25	170.64 ± 30.45	162.88–178.40	**3.0**
F_A_D of 6%	7.77 ± 0.18	223.58 ± 35.22	214.61–232.55	**4.0**

Data are presented as means ± SD. CI = confidence interval, F_A_D = alveolar concentration of desflurane, and F_I_D = inspired concentration of desflurane.
